# ProBDNF Upregulation in Murine Hind Limb Ischemia Reperfusion Injury: A Driver of Inflammation

**DOI:** 10.3390/biology12070903

**Published:** 2023-06-24

**Authors:** Katherine Aby, Ryan Antony, Yifan Li

**Affiliations:** Department of Basic Biomedical Science, Sanford School of Medicine, University of South Dakota, Vermillion, SD 57069, USA; katherine.aby@coyotes.usd.edu (K.A.); ryan.m.antony@coyotes.usd.edu (R.A.)

**Keywords:** skeletal muscle, brain-derived neurotropic factor, proBDNF, inflammation, ischemia-reperfusion, hind limb, myokine

## Abstract

**Simple Summary:**

Ischemia reperfusion injury in skeletal muscle is common, yet the underlying mechanisms are not well understood. Additionally, the role of brain-derived neurotrophic factor and its precursor in skeletal muscle have not been determined. To address this, the present study aimed to investigate the role of pro-brain-derived neurotrophic factor in ischemia reperfusion injury of the hind limb. It was shown that pro-brain-derived neurotrophic factor is significantly upregulated in skeletal muscle following injury, and that in the absence of skeletal muscle pro-brain-derived-neurotrophic factor signaling, the inflammatory response was significantly blunted. The overall conclusion is that pro-brain-derived neurotrophic factor is essential to the inflammatory response following ischemia reperfusion injury.

**Abstract:**

Brain-derived neurotropic factor (BDNF) has been shown to be expressed in many nonneuronal tissues including skeletal muscle. Skeletal muscle BDNF has been studied regarding its function in metabolism and exercise; however, less is known about its role in skeletal muscle injury. The precursor to BDNF, proBDNF, has an unknown role in skeletal muscle. The levels of proBDNF, mature BDNF, and their receptors were compared in the skeletal muscle and brain tissues of C57BL/6J mice. Tourniquet-induced hind limb ischemia-reperfusion injury was used to assess the function of skeletal muscle-derived proBDNF in skeletal muscle injury. Skeletal muscle-specific knockout of BDNF and pharmacological inhibition of p75NTR, the proBDNF receptor, were used to determine the role of proBDNF–p75NTR signaling. We show for the first time that proBDNF is the predominantly expressed form of BDNF in skeletal muscle and that proBDNF is significantly upregulated in skeletal muscle following hind limb ischemia-reperfusion injury. Skeletal muscle-specific knockout of BDNF blunted the inflammatory response in the injured tissue and appears to be mediated by the proBDNF–p75NTR pathway, as shown by the pharmacological inhibition of p75NTR. These findings suggest that skeletal muscle proBDNF plays a critical role in driving the inflammatory response following skeletal muscle injury.

## 1. Introduction

Brain-derived neurotrophic factor (BDNF), as its name implies, was originally discovered in the brain in the 1980s [1]. More recently, BDNF has been shown to be expressed in various peripheral tissues including the lungs, heart, spleen, and skeletal muscle [2,3,4,5,6,7,8]. While the expression, function, and distribution of BDNF has been characterized in the brain extensively, studies of peripheral BDNF expression and function are incomplete, and at times contradictory. 

In neurons, BDNF, as with other neurotrophic factors, is initially processed as proBDNF then later cleaved in the Golgi by the protease furin yielding mature BDNF [1,9,10,11,12,13,14]. Originally, BDNF was thought to be only active in its cleaved, mature form; however, it has since been shown in neurons that proBDNF is also released and active [2,5,8,9,10,13,15]. Once released into the extracellular space, proBDNF can be cleaved by metalloproteases or plasmin, or remain as proBDNF [9,12,13,14]. While this is accepted in neurons, BDNF cleavage and secretion in skeletal muscle is not fully understood. 

Released proBDNF acts on receptors p75NTR and sortilin, eliciting activation of downstream signaling pathways that lead to outcomes distinct from that of mature BDNF signaling through the receptor TrkB [5,8,10]. For example, while the mature BDNF–TrkB pathway plays a neuroprotective role in the nervous system, as well as a significant role in long-term potentiation, proBDNF causes neuronal death and long-term synaptic depression [11,16]. In skeletal muscle, mature BDNF seems to play a role in skeletal muscle metabolism; however, the function of proBDNF is unknown [3,17,18,19,20,21]

As the largest tissue mass in the body, skeletal muscle has a high risk for injury following trauma and surgical procedures. Particularly, skeletal muscle ischemia-reperfusion (IR) injuries are common following trauma, peripheral artery disease, surgery, or tourniquet placement [22,23]. While the ischemic conditions cause hypoxic and nutrient-deficient conditions leading to tissue damage, reperfusion further exasperates this damage including oxidative stress and endothelial damage leading to inflammation [24,25]. In skeletal muscle, this can lead to muscle swelling, muscle necrosis, and compartment syndrome and may lead to more severe systemic complications [23,24,26]. 

Since the BDNF–TrkB pathway is known for its role in survival and protection and proBDNF–p75NTR in cell death in neurons, this study aimed to understand whether and how these pathways are involved in skeletal muscle IR injury.

## 2. Materials and Methods

Mouse Model: All experimental protocols and use of animals in this study were reviewed and approved by the University of South Dakota Institutional Animal Care and Use Committee (IACUC), following the NIH guidelines of animal use in research. Male and female C57BL/6J mice between the ages of 12 to 16 weeks of age were chosen at random. Mice with skeletal muscle-specific knockout of BDNF were developed by crossing C57BL/6J mice with floxed *BDNF*, purchased from Jackson Labs [21], with skeletal muscle-specific Cre transgenic mice driven by the myosin light chain 1 promoter Myl1^tm1(cre)sjb^/J (Jackson Labs). 

Hind limb Ischemia-Reperfusion Model: Male and female C57BL/6J mice between 12 to 16 weeks of age were subject to acute ischemia-reperfusion injury (IR). Forty animals were used and weighed between 20.1–37.4 g, with the mean weight of 30.4 and 24.4 for male and female mice, respectively. Mice were anesthetized using 3% isoflurane and 1 mg/kg of Buprenorphine SR was administered to control pain. A 1/8″ 3.2 mm heavy orthodontic rubber band was placed at hip level on right hind limb using a McGivney ligator applicator [27,28], leaving the left hind limb as the contralateral control. Based on a comparative study measuring the force produced by orthodontic rubber bands on varying diameter rods, it is expected that the force produced on male and female would not be significantly different [27]. The rubber band was left in place for 1 h and complete ischemia was confirmed using laser Doppler imaging (Moor). One hour of ischemia was chosen for its ability to induce the inflammatory response without causing severe necrosis or delayed recovery. Following 1 h of ischemia, the rubber band was removed allowing reperfusion of the limb for 1–7 days. Following the reperfusion period, animals were sacrificed and tissue was collected from the IR-injured and contralateral control limbs.

LM11A-31 Treatment: Wildtype mice were either treated with 50 mg/kg of LM11A-31 diluted in drinking water or given regular drinking water [29,30,31]. Treatment began immediately following IR injury and continued until tissue was collected. 

Immunofluorescent staining: Tibialis anterior muscles were collected, coated in an optimal cutting temperature (OCT) compound, snap frozen in 2-methylbutane, and placed in a tray of OCT and kept at −80 °C for sectioning. Sections were cut to 10 μm using a Leica cryostat set at −18 °C and placed on charged slides. Frozen muscle sections were washed at room temperature in PBST for 5 min, fixed with 4% paraformaldehyde (PFA) for 10 min, and rinsed with PBST again for 5 min. Sections were microwaved in 10% tri-sodium citrate (pH 6.0) for 20 min maintaining a temperature of 90–100 °C. After 20 min, slides were allowed to cool to room temperature in the tri-sodium citrate followed by 3 × 5 min PBST washes. Sections were incubated with primary antibodies at 4 °C overnight. Following incubation, slides were washed in PBS 3 × 5 min, mounted with coverslips using Fluoromount mounting solution, and imaged using a Confocal (Olympus Fluoview 500, Center Valley, PA) or Olympus D microscope. Images were quantified using Image J, in which the total number of CD68+ or CD206+ cells were normalized to the total number or nuclei present as determined by counterstaining with Hoechst. To do this, channels were split so that each channel could be quantified individually; then, the number of puncta exceeding the predetermined pixel value were counted. Formal statistical analysis was then conducted using GraphPad Prism 9.

Western blot: Western blot was conducted as described in our previous publications [32]. Briefly, soleus, EDL, and brain samples were collected, snap frozen, and held at −80 °C until processed. Samples were lysed in 1× RiPA buffer supplemented with phosphatase inhibitor cocktail, protease inhibitor cocktail, and proteasome inhibitor MG132 using pestles in 1.5 mL tubes. Protein concentrations of the samples were equalized as determined using a Pierce BCA assay. Protein samples were subjected to standard SDS-PAGE electrophoresis and blotted using primary antibodies. Western blots were visualized using appropriate secondary antibodies conjugated with fluorescent dyes and LiCor scanner. Primary antibodies used were p75NTR (1:1000 Cell Signaling Technologies, 8238S), TrkB (1:1000, ProteinTech, 13129- 1-AP), sortilin (1:1000, ProteinTech, 12369-1-AP), proBDNF (1:500 Santa Cruz, sc-6551), BDNF (1:1000 Abcam, ab108319), Furin (1:1000 ABclonal, A7445), tPA (1:1000 ABclonal, A4210), MCP-1 (1:1000 Cell Signaling Technology, 2029S), IFN-γ (Cell Signaling Technology, 3159S), TNFα (1:500 Santa Cruz, sc-8301), and TGF-β (1:1000 Biolegend, 141402). Fluorescent dye 2,2,2-trichloroethanol was added to acrylamide gel prior to polymerization. Following electrophoresis, total protein loaded was imaged using UV gel imaging system. The total protein bands were then used to normalize the proteins blotted for (ratio to control) [33].

PCR: The plantaris muscle from both the IR and control limbs are collected and homogenized using pestles in 1.5 mL test tubes containing 400 μL of RNA-zol. Samples are processed according to the manufacturer’s instructions (Zymo Research Direct-zol RNA miniprep kit). Final RNA product is analyzed for purity and concentration using Thermo Scientific Nanodrop 2000. RNA is then transcribed into cDNA using Applied Biosystems Multiscribe high-capacity cDNA kit according to the manufacturer’s instructions. The cDNA is then used in conventional PCR using Promega GoTaq master mix. Primer sequences used were BDNF1-F GTGTGACCTGAGCAGTGGGCAAAGGA, BDNF2-F GGAAGTGGAAGAAACCGTCTAGAGCA, BDNF3-F GCTTTCTATCATCCCTCCCCG AGAGT, BDNF4-F CTCTGCCTAGATCAAATGGAGCTT C, BDNF5-F CTCTGTGTAGTTTCATTGTGTGTTC, BDNF6-F GCTGGCTGTCGCACGGTTCCCATT, BDNF7-F CCTGAAAGGGTCTGCGGAACTCCA, BDNF8-F GTGTGTGTCTCTGCGCCTCAGTGGA, BDNF9A-F CCCAAAGCTGCTAAAGCGGGAGGAAG, BDNFcds-R GAAGTGTACAAGTCCGCGTCCTTA, PC1/3 F CTCTCCCAGTGAGCCTCTAGC, PC1/3 R TAGTGCACACCAAACGCAAA. PC2 F TCCACACACCCGCAGTTTT, PC2 R TGGCCCCTTCTCAAACAGTG, PC4 F ACCCTGGGCCTGGAGAATAA, PC4 R GTAGTAGTACAGAGTTCCTGGGC, PC5 F CCCGAGGGTATGTCAAGGGT, PC5 R CTAAGCAGTCAAAGGAGGTGGT, PC6 F GTGGATGCAGAAGCTCTGGT, PC6 R AGCTGAAGGTGCGATATGGG, Furin F TGGAAAGCTACTGCCTGACG, Furin R GGA TGGAGACCACAATGCCA, 18S RNA F AGTCCCTGCCCTTTGTACACA, and 18S RNA R CGATCCGAGGGCCTCACTA.

ELISA: ELISA assays were conducted with 10 μL of whole muscle lysate from the soleus muscles of IR and contralateral control limbs lysed with 1× RiPA buffer supplemented with phosphatase inhibitor cocktail, protease inhibitor cocktail, and proteasome inhibitor MG132 according to the manufacturer’s instructions (Biolegend ELISA Max). The IR limb was normalized to the control limb and the data were analyzed over the time course of IR. 

Statistical analysis: Data are presented as mean ± standard deviation (SD). One-way ANOVA followed by Tukey’s post hoc test or student’s *t* test were applied using GraphPad Prism 9 software. Differences were considered statistically significant at *p* < 0.05.

## 3. Results

### 3.1. BDNF Exon VI Splice Variant Is Expressed by Skeletal Muscle

**Figure 1:**
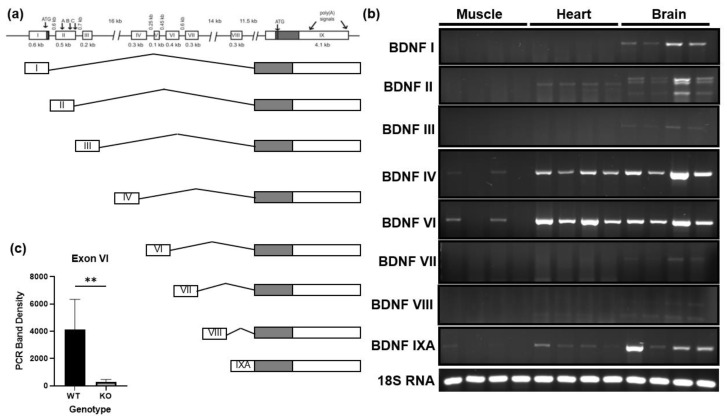
Splice variant expression of BDNF gene in skeletal muscle, heart, and brain of C57BL6J mice. (**a**) Layout of murine BDNF gene and splice variants adapted from Aid et al. [34]. (**b**) Representative agarose gel images comparing mRNA expression of reported splice variants of the murine BDNF gene in brain, heart, and gastrocnemius muscle samples. (**c**) Quantification of exon VI in wildtype (WT) and BDNF skeletal muscle knockout (KO) using a student’s *t*-test; *n* = 6, ** *p* < 0.01.

It has been reported that the murine BDNF gene can be alternatively spliced, resulting in 11 unique splice variants, and that various regions of the brain as well as various tissues are unique to the variants they express [34,35]. These splice variants only vary in the untranslated region of the gene as they all include the same 3′ pre-proBDNF coding region which is highly conserved between species. Using primers specific to reported murine BDNF mRNA splice variants, we compared the variants expressed in the gastrocnemius, heart, and whole brain tissues [34,35]. Figure 1a illustrates the structure of the murine BDNF gene and each of the reported splice variants. Figure 1b shows that the brain expressed many of the splice variants as expected. In comparison, skeletal muscle primarily expressed BDNF exon VI splice variant. This was consistent with the splice variants observed in the heart, though the heart expressed additional variants including the exon II splice variant. In samples from animals with the skeletal muscle-specific knockout of BDNF, shown in lanes 2 and 4 in Figure 1b, the expression of BDNF exon VI was significantly reduced compared to wildtype littermates (Figure 1c), confirming that the source of the detected BDNF VI is skeletal muscle.

### 3.2. Comparison of BDNF Protein Levels in Skeletal Muscle and Brain

**Figure 2:**
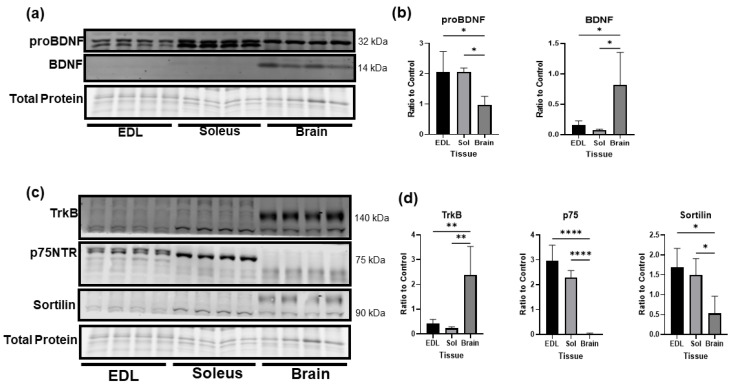
Comparison of protein levels of BDNF, proBDNF, and their receptors in skeletal muscle and brain. (**a**) Representative Western blot images comparing protein levels of proBDNF and mature BDNF in fast twitch (EDL) and slow twitch (soleus) skeletal muscle compared to the brain. (**b**) Quantification of Western blot densitometry of proBDNF and mature BDNF as a ratio to total protein loading control compared using one-way ANOVA followed by Tukey’s post hoc test *n* = 4 per group, * p < 0.05. (**c**) Representative Western blot images comparing protein levels of receptors TrkB, p75NTR, and sortilin in fast twitch (EDL) and slow twitch (soleus) skeletal muscle compared to the brain. (**d**) Quantification of Western blot densitometry of TrkB, p75NTR, and sortilin as a ratio to total protein loaded and compared using one-way ANOVA followed by Tukey’s post hoc test *n* = 4 per group, * *p* < 0.05, ** *p* < 0.01, **** *p* < 0.0001.

The cleavage of BDNF has been well studied in neurons but is essentially unknown in skeletal muscle. In Figure 2a, we compared the levels of proBDNF and mature BDNF in fast twitch (EDL) and slow twitch (soleus) skeletal muscle and whole brain lysate. While the brain expressed significantly more mature BDNF than skeletal muscle, skeletal muscle expressed significantly higher levels of proBDNF compared to the brain under baseline conditions (Figure 2b). Further, the expressions of p75NTR and sortilin, the receptors for proBDNF, were significantly higher in both slow and fast twitch muscle fibers than in the brain. In contrast, the brain had significantly higher expression of TrkB, the receptor for mature BDNF (Figure 2c,d). Overall, these data clearly indicate that, different from the brain in which the mature BDNF–TrkB pathway is predominant, the proBDNF–p75NTR pathway is the predominant pathway in skeletal muscle.

### 3.3. Comparison of the Levels of Proprotein Convertases in Skeletal Muscle and Brain

**Figure 3:**
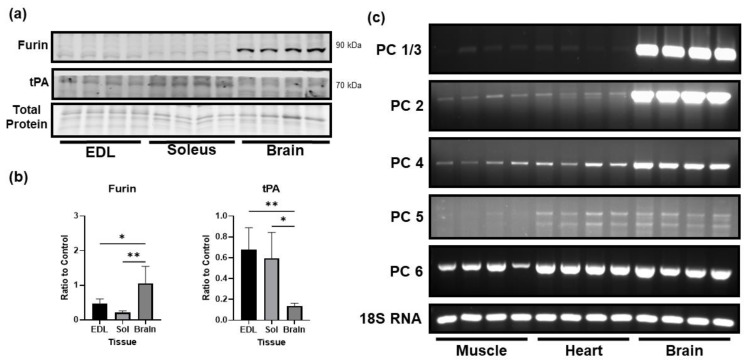
Low levels of proBDNF in skeletal muscle may be a consequence of low levels of pro-protein convertases. (**a**) Representative Western Blot images of furin and tissue plasminogen activator (tPA). (**b**) Quantification of Western blot images for furin and tPA normalized to total protein loading control and compared using one-way ANOVA analysis followed by Tukey’s post hoc test, *n* = 4, * *p* < 0.05, ** *p* < 0.01. (**c**) Representative agarose gel images comparing mRNA expression of alternative proprotein convertases in skeletal muscle (gastrocnemius), heart, and brain.

In the brain, proBDNF is primarily cleaved to mature BDNF by the proprotein convertase furin. We compared the protein level of furin and other convertases in skeletal muscle and brain tissue. Clearly, the protein level of furin was significantly lower in the skeletal muscle as compared to the brain (Figure 3a,b). Additionally, levels of mRNA expression of other major proprotein convertases were also lower in skeletal muscle compared to the brain (Figure 3c). Interestingly, we did find that skeletal muscle expressed significantly higher levels of tissue plasminogen activator (tPA) compared to the brain (Figure 3a,b). This result suggests that the skeletal muscle has a potential to cleave proBDNF extracellularly. However, given the higher level of proBDNF in skeletal muscle, the role of plasmin in control of proBDNF and mature BDNF in skeletal muscle remains to be determined. 

### 3.4. BDNF Is More Highly Expressed in Young Developing Skeletal Muscle

**Figure 4:**
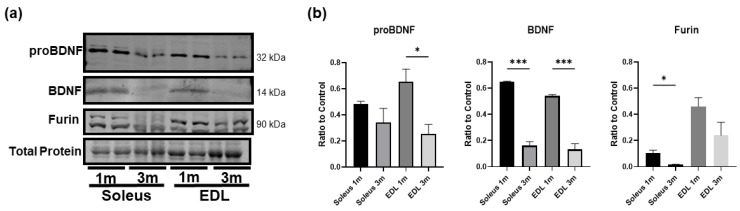
BDNF is more highly expressed in the skeletal muscle of younger animals compared to adult animals. (**a**) Representative Western blot images of proBDNF, BDNF, and furin in the soleus and EDL of 1 month old (1 m) and 3-month-old (3 m) mice. (**b**) Quantification of Western blot densitometry as a ratio to total protein loading control and compared using one-way ANOVA analysis followed by Tukey’s post hoc test; *n* = 4, * *p* < 0.05, *** *p* < 0.001.

Since mature BDNF is critical in brain development and growth, we asked whether skeletal muscle mature BDNF level would be greater in early age. To this end, we compared the BDNF protein expression in the soleus and EDL of 1-month-old and 3-month-old mice. The results show that in both the soleus and EDL, BDNF and proBDNF levels were significantly higher in the young 1-month-old mice compared to the 3-month-old mice (Figure 4a,b), and the level of mature BDNF showed a clear decline with age, while proBDNF protein expression also seems to decline with age. Moreover, the protein expression of furin was significantly reduced in the soleus of 3-month-old mice compared to the younger mice. These results suggest that mature BDNF may be functionally important in early muscle growth and development but less so in adult muscle. As a result, mature BDNF level declines and proBDNF becomes the predominant form in adult muscle.

### 3.5. IR Injury Significantly Upregulates ProBDNF in Injured Tissue

**Figure 5:**
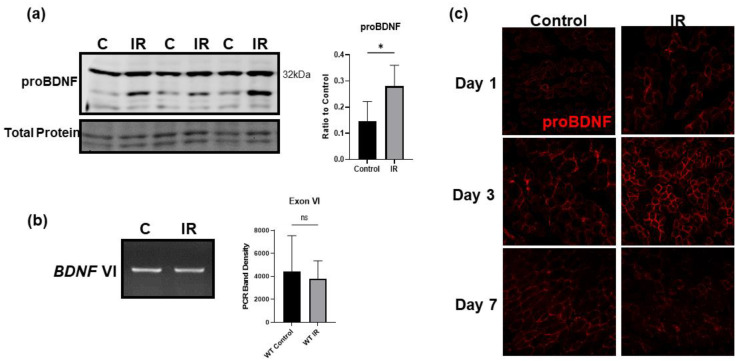
IR injury significantly upregulates proBDNF in injured tissue. (**a**) Representative Western blot of proBDNF upregulated in IR tissue compared to contralateral control (C) tissue and quantification of Western blot densitometry as a ratio to total protein loading control and compared using Student’s *t* test; *n* = 6, * *p* < 0.05. (**b**) Representative agarose gel image comparing mRNA expression of BDNF exon VI between control and IR-injured gastrocnemius muscle and corresponding quantification using a Student’s *t* test; *n* = 3 ns = not significant. (**c**) Representative immunofluorescent images showing proBDNF in contralateral control and IR-injured tissue on reperfusion days 1, 3, and 7.

Using a model of tourniquet-induced IR injury of the hind limb, we found that following IR injury proBDNF is significantly upregulated in the IR-injured tissue both in soleus, a type-I fiber-predominant muscle, and EDL, a type-II fiber-predominant muscle (Figure 5a). The upregulation of proBDNF was further confirmed using immunofluorescent staining comparing the contralateral control tissue to the ischemic tissue shown in Figure 5c. Across the time course of IR injury at days 1, 3, and 7 of reperfusion, the greatest increase in proBDNF occurred on day 3 of reperfusion. Noticeably, BDNF exon VI mRNA levels remain unchanged in ischemic tissue compared to control (Figure 5b), indicating that the changes in the proBDNF are likely due to changes in post translational modification or decreased degradation of the protein rather than increased gene expression. We also see that in the immunofluorescent staining of proBDNF, it appears that proBDNF is slightly upregulated in the contralateral control tissue at day 3 of reperfusion. This may reflect the stimulation of skeletal muscle proBDNF expression by circulating factors such as cytokines and/or chemokines released from the site of injury.

### 3.6. IR-injured Muscle Induces a Significant Inflammatory Response

**Figure 6:**
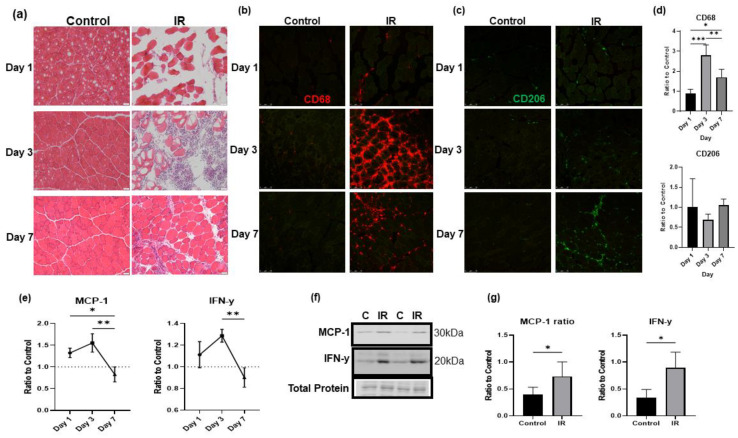
IR injury-induced inflammatory responses. (**a**) H&E staining showing the progression of IR injury in the tibialis anterior muscle (IR) compared to the contralateral limb (control) on reperfusion days 1, 3 and 7. (**b**) Immunostaining for CD68-positive cells in the IR-injured tibialis anterior (IR) compared to the contralateral control limb (control) on reperfusion days 1, 3, and 7. (**c**) Immunostaining for CD206-positive cells in the IR-injured and contralateral tibialis anterior muscles on reperfusion days 1, 3, and 7. (**d**) Quantification of CD68 and CD206 staining comparing the number of puncta in IR images to their contralateral controls; significance was determined by one-way ANOVA followed by Tukey’s post hoc test, *n* = 4 per group, * *p* < 0.05, ** *p* < 0.01, *** *p* < 0.001. (**e**) ELISA assays showing a peak in inflammatory cytokines MCP-1 and IFN-γ at reperfusion day 3 compared to the level of the contralateral control; significance was determined by one-way ANOVA followed by Tukey’s post hoc test, *n* = 4 per group, * *p* < 0.05, ** *p* < 0.01. (**f**) Representative Western blot images showing increased MCP-1 and IFN-γ in IR-injured tissue compared to contralateral controls (C). (**g**) Quantification of Western blot images in panel (**f**) as a ratio to total protein loading control and compared using student’s *t* test; * *p* < 0.05, *n* = 6.

Following tourniquet-induced IR injury, there was significant damage to the skeletal muscle fibers as well as a massive infiltration of nucleated cells into the injured tissue. The increase in infiltrated cells peaked around day 3 of IR injury and was followed by the resolution and recovery phase, as seen in the H&E-stained TA muscle in Figure 6a. By day 7 of reperfusion, the tissue was significantly recovered as seen by large, closely packed muscle fibers and reduced number of infiltrating cells. Immunofluorescent staining of CD68, a marker for proinflammatory macrophages, and CD206, a marker for anti-inflammatory macrophage populations, revealed dynamic changes in inflammation during reperfusion. A peak in staining of CD68 positive cells appeared on day 3 of reperfusion, and a peak in staining of CD206 positive cells appeared on day 7 of reperfusion as seen in Figure 6b,d. This change was further confirmed by the results of ELISA assays for MCP-1 and IFN-γ in Figure 6e. These pro-inflammatory chemokine and cytokine slightly increased on day 1 compared to control, further increased on day 3, and then reduced below the control level by day 7 of reperfusion. In Figure 6f,g, Western blot analysis also showed significant increases in these inflammatory cytokines in IR-injured tissue compared to the contralateral control limb. Overall, these results confirmed that tourniquet-induced skeletal muscle IR injury exhibits a typical pattern of inflammation, inflammation resolution, and recovery sequences over a one-week period. Notably, the peak in pro-inflammatory response was consistent with the peak of proBDNF upregulation, suggesting that proBDNF may be involved in the inflammatory response.

### 3.7. Skeletal Muscle-Specific Knockout of BDNF Attenuates Proinflammatory Response following IR Injury

**Figure 7:**
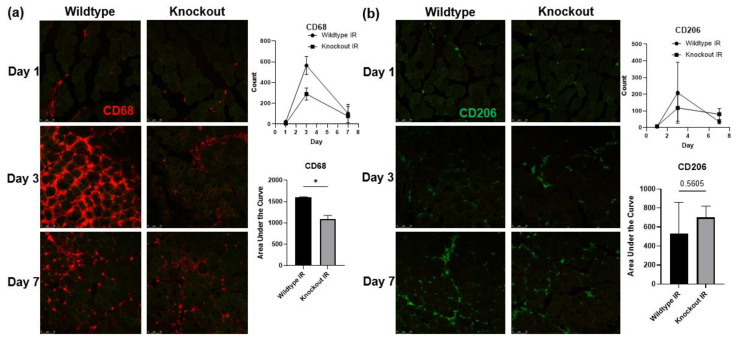
Immunofluorescent staining showing the infiltration of immune cells into IR-injured tissue. (**a**) Staining for CD68 positive cells in the IR-injured tibialis anterior in wildtype compared to BDNF skeletal muscle knockout animals on reperfusion days 1, 3, and 7, and corresponding quantification using counts of puncta over time and analyzed using area under the curve analysis *n* = 3 per group; * *p* < 0.05. (**b**) Staining for CD206 positive cells in the IR-injured tibialis anterior in wildtype compared to BDNF skeletal muscle knockout animals on reperfusion days 1, 3, and 7, and corresponding quantification of CD206 positive puncta in IR; significance was determined by area under the curve analysis, *n* = 3 per group, significance set at *p* < 0.05.

**Figure 8:**
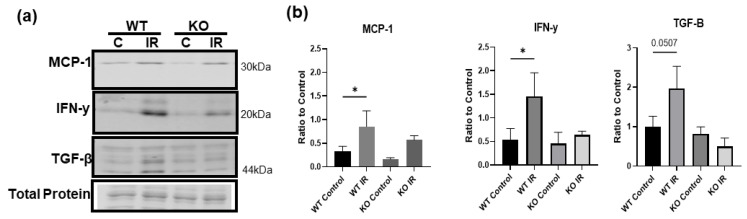
Inflammatory proteins are decreased in ischemic injury in knockout animals. (**a**) Representative Western blot images showing MCP-1, IFN-y, and TGF-B in wildtype (WT) and knockout (KO), control (C), and IR tissue. (**b**) Quantification of Western blot densitometry analyzed as a ratio to total protein loading control and compared using one-way ANOVA analysis *n* = 3, * *p* < 0.05.

To test whether the upregulated proBDNF is involved in the inflammatory response in IR-injured skeletal muscle, mice with skeletal muscle-specific BDNF knockout were used. PCR analysis of BDNF expression in knockout animals showed a 93% reduction in BDNF exon VI compared to BDNF-floxed animals (data not shown). Immunofluorescent staining for proinflammatory CD68+ macrophages (Figure 7a) showed that compared to wildtype mice, BDNF knockout mice showed a significant reduction in the infiltration of CD68+ macrophages in the injured muscle. Concurrently, wildtype and BDNF knockout mice showed similar populations of CD206+ anti-inflammatory macrophage populations (Figure 7b). These data indicate that the proinflammatory macrophage polarization was blunted in the absence of proBDNF, while the anti-inflammatory phenotype was unaffected. Consistently, in wildtype animals, pro-inflammatory chemokine MCP-1 and cytokine IFN-y were significantly increased in the IR-injured muscle compared to the contralateral control muscle; however, these increases were diminished in the BDNF knockout animals (Figure 8a,b), further indicating that proBDNF plays a role in driving inflammation following IR injury.

### 3.8. p75NTR Inhibition Attenuates the Proinflammatory Response following IR Injury

**Figure 9:**
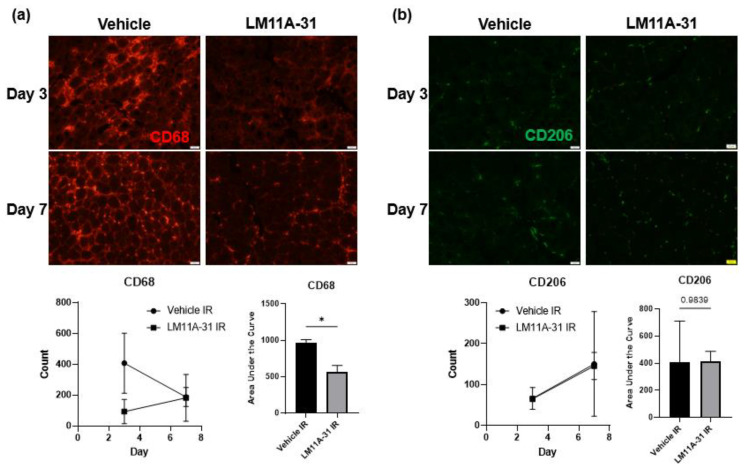
Immunofluorescent staining showing the infiltration of immune cells into IR-injured tissue in vehicle and LM11A-31 treated animals. (**a**) Staining for CD68-positive cells in the IR-injured tibialis anterior in vehicle compared to LM11A-31 treated animals on reperfusion days 1, and 3, and corresponding quantification using counts of puncta over time and analyzed using area under the curve analysis *n* = 3; * *p* < 0.05. (**b**) Staining for CD206-positive cells in the IR-injured tibialis anterior in in vehicle compared to LM11A-31 treated animals on reperfusion days 1 and 3, and corresponding quantification of CD206 positive puncta in IR; significance was determined by area under the curve analysis, *n* = 3 per, significance set at *p* < 0.05.

To test whether proBDNF affects IR injury-induced inflammation via its receptor p75NTR, a pharmacological inhibitor of p75NTR, LM11A-31, was used [30,31]. Animals undergoing treatment with LM11A-31 (50 mg/kg/day) were subjected to IR injury [29]. At the end of treatment, the tibialis anterior muscles were stained with the proinflammatory macrophage marker CD68. It was seen that in the treatment group the number of CD68+ cells were significantly reduced in the injured muscle compared to the vehicle-treated controls (Figure 9a). Similarly, it was seen that the treatment group had similar numbers of anti-inflammatory macrophages as vehicle-treated animals (Figure 9b). These results further support the notion that it is proBDNF that is responsible for driving the proinflammatory response following IR injury.

## 4. Discussion

By characterizing gene expression variants, protein forms, and function of BDNF in skeletal muscle in IR injury, this study advances our understanding of skeletal muscle-derived BDNF in several ways. First, we compared BDNF variant expression in the brain, heart, and skeletal muscle, and our data show that variant VI is the primary expressing BDNF variant in skeletal muscle in C57BL/6J mice. While the findings of expressed variants are consistent with those from Aid et al. [34] in the brain and heart, they contradict their finding that the dominantly expressed variant in skeletal muscle was *BDNF* VIII. This contradiction could be due to differences in mouse lines and muscle types. Further work is needed to thoroughly investigate whether different muscles express different variants depending on composition of muscle fiber type, location, and functional conditions. It is also possible that the different mouse lines express different splice variants. To our knowledge this is the first time the BDNF exon VI has been shown to be the predominant splice variant expressed in C57BL/6J mice gastrocnemius muscle. In addition to the gene variants, posttranslational processes are critical for BDNF activity. Different functions of the mature BDNF–TrkB pathway and the proBDNF–p75NTR pathway have been well characterized in neurons. In comparison, although BDNF is well accepted as a myokine [4,5,6,8], BDNF forms and their functions in skeletal muscle are poorly studied. To our knowledge, this is the first study to compare the differences of mature BDNF and proBDNF between skeletal muscle and brain. Our data show that skeletal muscle expresses much higher levels of the uncleaved proBDNF compared to the brain, while the brain expresses much higher levels of mature BDNF. As evidenced by our study, the predominance of proBDNF in skeletal muscle is likely due to low levels of proprotein convertases in skeletal muscle. These findings raise important questions: is BDNF expression and processing different in skeletal muscle compared to the brain, and what is its functional significance? These differences could be related to the functioning of brain and skeletal muscle. Neurons and skeletal muscles are very different in many ways. Neurons have constantly high activities and metabolic rates, which may rely heavily on the role of the mature BDNF–TrkB pathway to maintain cell survival and synapse integrity. In comparison, skeletal muscle varies significantly depending on physical and environmental condition and has high risk for injury and disuse, leading to atrophy and breakdown of proteins, in which proBDNF and its downstream pathways may play an important role. This is also supported by the fact that BDNF is more highly expressed in young developing skeletal muscle compared to aging adult skeletal muscle, where muscle growth and innervation may rely more on mature BDNF–TrkB activities. 

The notion that skeletal muscle proBDNF plays a significant role in injury is supported by our functional studies. In IR-injured muscle, proBDNF was significantly upregulated. The time course of proBDNF changes were consistent with the course of the inflammatory response. Moreover, skeletal muscle knockout of BDNF or blockage of proBDNF receptor p75NTR blunted IR injury-induced inflammation. These results suggest that upregulated proBDNF in injured skeletal muscle promotes inflammation. Inflammation is critical for clearing dead cells and stimulating tissue regeneration. On the other hand, uncontrolled and persistent inflammation is detrimental and inhibitory for injury repair and functional recovery. Thus, it is important to understand the underlying mechanisms by which skeletal muscle proBDNF regulates inflammatory processes during IR injury. In the present study, we show evidence that proBDNF may play a role in the inflammatory response. Specifically, our data show that the course of proinflammatory macrophage changes follows the changes of proBDNF and was blunted by BDNF knockout and p75NTR inhibition, suggesting that skeletal muscle proBDNF may regulate macrophage infiltration or inflammatory polarization. At this time, it is not clear whether proBDNF released from the injured skeletal muscle directly regulates macrophage functions via p75NTR in macrophages, or if the proBDNF–p75NTR pathway in skeletal muscle controls the release of chemokines or other myokines that regulates macrophage activities. This is potentially an important mechanism, which needs to be thoroughly investigated. Additionally, it is unclear if the blunting of the inflammatory response by BDNF knockout and p75NTR inhibitions is entirely beneficial. As mentioned above, while chronic inflammation is detrimental to health, acute inflammation is necessary to properly activate the recovery process. It is possible that skeletal muscle proBDNF–p75NTR-mediation of acute inflammation is essential for injury healing and recovery, which needs to be further investigated.

Our data clearly indicates that proBDNF is the predominant form of BDNF expressed by skeletal muscle and that it plays a role in IR injury and inflammation in skeletal muscle. However, this study did not test and cannot rule out the role of mature BDNF in skeletal muscle injury and inflammation. However, as our data show, the levels of mature BDNF and TrkB receptors in skeletal muscle are low. Moreover, there was a trend of downregulation of mature BDNF along with the upregulation of proBDNF at the early phase of IR injury and inflammation. Therefore, it is possible that the mature BDNF–TrkB pathway may not play a significant role in the early IR injury and inflammatory response. However, it is not currently clear as to whether mature BDNF is critical in the later regeneration phase. Nevertheless, it is important to understand the balance between pro and mature BDNF following skeletal muscle injury to be able to develop targeted treatments. Further investigation into the driving mechanisms behind the proBDNF-mature BDNF balance is necessary to fully understand how to target them for treatment. 

## 5. Conclusions

Compared to the brain which expresses high levels of BDNF, skeletal muscle expresses higher levels of proBDNF and comparably lower levels of mature BDNF. Skeletal muscle predominately expresses the exon VI splice variant compared to the brain which expresses nearly all the reported variants. While myokine BDNF has been investigated with regards to skeletal muscle metabolism, here, we show that myokine BDNF also plays a major role in tourniquet-induced IR skeletal muscle injury. Our data show that the inflammatory conditions following skeletal muscle injury are regulated by the BDNF. This is likely due to the BDNF precursor, proBDNF, based on the high level of proBDNF and p75NTR expressed in skeletal muscle and the relatively low level of mature BDNF, TrkB, and protein convertases. When BDNF is knocked out in skeletal muscle or when the p75NTR signaling is blocked, there is a significant reduction in inflammatory response, indicating that the proBDNF–p75NTR pathway is indeed important in driving inflammation in skeletal muscle IR injury. 

## Data Availability

Data are contained within the article.
